# Undiagnosed AIDS in a 13-year-old boy in rural Gabon

**DOI:** 10.1016/j.idcr.2024.e02103

**Published:** 2024-10-23

**Authors:** Saskia Dede Davi, Ayodele Alabi, Lillian Rene Endamne, Teite Rebecca Hildebrandt, Anita Lumeka, Dearie Glory Okwu, Rella Zoleko-Manego, Ghyslain Mombo-Ngoma, Selidji Todagbe Agnandji, Michael Ramharter

**Affiliations:** a Center for Tropical Medicine, Bernhard-Nocht Institute for Tropical Medicine & I. Dept. of Medicine, University Medical Centre Hamburg-Eppendorf, Hamburg, Germany; b German Centre for Infection Research, Partner Site Hamburg-Lübeck-Borstel-Riems, Hamburg-Lübeck-Borstel-Riems, Germany; c Centre de Recherches Médicales de Lambaréné, Lambaréné, Gabon; d Leiden University Center for Infectious Diseases (LU-CID), Leiden University Medical Center, Leiden, the Netherlands; e Institute of Tropical Medicine, Travel Medicine and Human Parasitology, Competence Centre for Tropical Medicine Baden-Württemberg, Universitätsklinikum Tübingen, Tübingen, Germany; f German Center for Infectious Diseases Research (DZIF), Tübingen, Germany; g Department of Implementation Research, Bernhard-Nocht Institute for Tropical Medicine, Hamburg, Germany

**Keywords:** HIV, Mother-to-child-transmission, Paediatric HIV/AIDS, Pregnancy, Gabon

## Abstract

**Introduction:**

To date, 38.4 million people live with the Human Immunodeficiency virus (HIV) amongst whom 1.7 million are children below fourteen years of age. The highest burden of HIV is in sub-Saharan Africa. Children living with HIV acquired the infection mostly by mother-to-child transmission (MTCT), however the diagnosis is often delayed.

In malaria-endemic countries, fever is one of the most frequent symptoms for seeking medical care and it is often primarily suspected as the onset of malaria or respiratory bacterial infections. Here, we report a case of late-onset undiagnosed AIDS in a 13-year-old boy living in rural Gabon in the Gabonese tropical rainforest in the province of Ngounié.

**Case:**

A 13-year-old orphan child presented at our routine consultation for fever screening at the Institut de Santé de Sindara (ISSA) in 2021 due to remittent fever episodes, paleness, chronic fatigue and cough. His medical history documented repeated consultations and hospitalisations over the past years, establishing various diagnoses and treatments without significantly improving his condition. Serologic testing established the diagnosis of HIV-1 infection, classifying it as CDC stage 3 AIDS. Given the family history, late-onset symptomatic HIV infection 13 years after mother-to-child transmission was the most likely transmission mode.

**Discussion:**

HIV infection may occur in older children and young adolescents and should be considered as an important differential diagnosis of reappearing fevers in regions of malaria transmission. Early diagnosis of HIV, particularly in children and adolescents, improves health outcomes. highlighting the need for HIV testing in children and adolescents.

## Introduction

Currently, 38.4 million people live with the Human Immunodeficiency virus (HIV). Among those, 1.7 million are children below fourteen years of age. The brunt of the global burden of HIV infection is in sub-Saharan Africa [1].

According to UNAIDS, approximately 15 % of all HIV-infected individuals are unaware of their HIV status [1]. In Gabon, approximately 3 % of the population is infected with HIV [1]. In the fight against the spread of HIV infection and to reduce mother-to-child transmission (MTCT) of HIV, Gabon established a national HIV/AIDS control programme with systematic screening for all pregnant women attending the antenatal care (ANC) unit in 2009. Women diagnosed with HIV receive immediate antiretroviral treatment (ART) offered cost-free by the national HIV/AIDS control programme [2]. These efforts have helped to reduce MTCT of HIV [2]. However, ANC services are usually based in semi-urban and urban regions in Gabon and may not be accessible to all residents of more remote regions. Here, we report a case of late-onset AIDS in a 13-year-old boy living in a village amid the Gabonese tropical rainforest in the province of Ngounié.

## Case

From August 2021 to November 2021, a prospective cross-sectional study was done to determine the prevalence of Coronavirus disease 2019 (COVID-19) and *Plasmodium falciparum* infection in rural Gabon. All individuals at least four years of age who attended the mobile diagnostic test services were invited to participate in this study. During this period, a 13-year-old Gabonese boy presented with cough, weakness, fatigue and eyelid oedema to the outpatient department of the Institut de Santé de Sindara (ISSA), a sentinel site of the Centre de Recherches Médicales de Lambaréné (CERMEL) based in Sindara. He complained about remittent fever of more than 38 °C, rhinorrhoea, cough, dyspnoea, diarrhoea, headache, loss of appetite and weakness for more than six months. The patient had been experiencing recurrent episodes of these symptoms since 2019. He sought medical care several times in five different facilities in two provinces (Moyen-Ogooué and Ngounié). During these encounters with the formal health care system, he had been repeatedly treated for suspected malaria, bacterial diarrhoea, bronchitis, pneumonia and bronchopneumopathy of unknown origin with dexamethasone, antibiotics (amoxicillin/clavulanic acid, cotrimoxazole), antihelminthics (albendazole), antimalarials (artemether/lumefantrine), and another antiparasitic (pyrimethamine). The patient had been admitted to the hospital more than six times within two years with the above-mentioned clinical symptoms.

In December 2019 the patient sought medical care with a prominent productive cough and fatigue. An X-ray revealed bilateral infiltration of the lungs with bilateral hilar lymph nodes.

The patient was hospitalised and treated with amoxicillin/clavulanic acid for suspected pneumonia. He received additional anti-malarial treatment with intravenous 250 mg of quinine sulfate. Despite these multiple therapies, the health status of the patient worsened.

In June 2020, he complained again of fever, paleness, and cough and splenomegaly and facial oedema were noted in the hospital records. A seven-month recurrent fever was diagnosed as a bronchopneumopathy of unknown origin. Tracing his medical history, no chronic diseases were mentioned but the patient had recurrent periods of sickness since childhood according to the grandmother. However, during consultation it was found out that the patient was an orphan as his mother and father died in 2020 and 2021, respectively, in their 20 s of unknown causes. At our institution the patient presented as a 13-year-old boy with a height of 126 cm and a weight of 29 kg – both being significantly below the average of his age, indicating inadequate physical development. His mucus membranes (conjunctiva), skin colour, hands and feet were pale. The patient had swollen eyes (periorbital oedema) and a swollen face (facial oedema). Swollen lymph nodes were palpable all over the body, indicative of generalised lymphadenopathy. There were disseminated crackles on the left lung lobe. The clinical examination of the heart was normal. The clinical abdominal examination showed a splenomegaly, previously described in May 2020. Ascites was clinically suspected due to abdominal distension.

Based on the clinical presentation, we suspected a nephrotic syndrome and a urine dipstick was performed. The urine was frothy and the dipstick demonstrated a slightly elevated specific urine weight at 1.025 g/mL, +++ for proteins, + for leucocytes, + for erythrocytes and no nitrite, suggestive of massive proteinuria.

To further investigate the febrile episodes and respiratory symptoms, a malaria rapid diagnostic test was done, and a PCR test for SARS-CoV-2 was performed, with both test results being negative. We referred the patient to the referral hospital for further treatment of the nephrotic syndrome and later found out that he was treated for severe malaria without laboratory evidence. Meanwhile, biological samples were further processed at our institution for haematology, biochemistry, microbiological analysis, and screening for common infectious diseases. However, differential diagnosis of several diseases potentially associated with nephrotic syndromes, such as autoimmune diseases and malignancies, could not be determined in this resource-limited setting.

The patient had severe anaemia with a haemoglobin of 3.6 g/l (normal range: 10,8 – 13,3 g/dl) and thrombocytopenia of 109.000/µl (normal range: 150.000 – 400.000 /µl). The lymphocyte count was 6.6 × 10^9^/L (normal range: 1–4 × 10^9^/L).

Biochemistry analysis showed a C-reactive protein level of 8.58 mg/l (normal range: < 5 mg/l). Alanine transaminase 7.3 U/l (10 − 35), total bilirubin 3.2 µmol/l (normal range: 5.13 – 23.95) and albumin 24.5 g/l (normal range: 26 – 56) levels below the normal range. The total protein was elevated at 83.5 g/l (normal range: 36 – 81.5 g/l). All the other parameters (aspartate transaminase, Magnesium, uric acid, creatinine, alkaline phosphatase, direct bilirubin, gamma-glutamyl transferase, glucose, phosphate) were normal. In addition, we quantified the proteinuria first detected by urine dipstick by determining 24/h urine. The patient had proteinuria in a nephrotic range (at 3 g/d.) A thick smear was performed to determine malaria and blood-borne filarial infections. There was no presence of *Plasmodium* spp. nor microfilariae. A stool analysis was done. The stool was green, normal smelling and soft. Further, stool microscopy was done using the Kato-Katz analysis ruling out soil-transmitted helminth infections. In addition, coproculture and Harada-Mori techniques were used to assess the presence of hookworm larvae or *Strongyloides stercoralis,* with both tests being negative. To determine the presence of protozoa, the Merthiolate-Iodine-Formaldehyde technique was used, and only the apathogenic *Blastocystis hominis* and *Entamoeba coli* were detected.

Based on the prolonged clinical symptoms associated with remittent fevers, generalised lymphadenopathy, bicytopenia, wasting and apparent nephrotic syndrome, we suspected HIV-associated nephropathy as the underlying cause of the clinical status of this paediatric patient.

Blood samples were analysed for HIV, hepatitis B virus (HBV) and hepatitis C virus (HCV). The assessment of HIV was done using three different rapid diagnostic tests, according to the Gabonese National HIV/AIDS Control Programme. The first test was Determine™ HIV-1/2 (Abbot, Illinois, US), which is based on antibodies. Then Determine™ HIV-1/2 AG/AB Combo (Abbot, Illinois, US), based on antibodies and antigens, was used. The third test was ABON HIV 1/2/O Tri-Line (ABON Biopharm, Hangzhou, China), used to differentiate and confirm whether he was either HIV 1 or HIV 2 positive. Following this algorithm, HIV-1 was diagnosed. The serology tests for HBV and HCV were negative. The CD4 testing indicated a count of 160 cells/µl. Morning sputum was collected to assess pulmonary tuberculosis using GeneXpert, which yielded a negative result.

Abdominal ultrasound demonstrated no pleural effusion or ascites and moderate splenomegaly. After establishing the diagnosis of HIV infection, CDC stage C3, complicated by HIV-associated nephropathy, we transferred the patient to the national HIV centre in Lambaréné, where ART with abacavir, lamivudine and dolutegravir was initiated and complemented with trimethoprim-sulfamethoxazole prophylaxis for the prevention of opportunistic infections. Viral load testing was unavailable at baseline, but three months after ART initiation, viral load was already effectively suppressed to about 215 copies/mL. During follow-up, the boy was in a good condition and did not report any complaints.

## Discussion

In Gabon, routine HIV testing for pregnant women was established in 2009 [2]. Our patient was born in 2008 and, therefore, before the start of this national HIV control programme. Thus, the patient has fallen off the grid of routine HIV testing. Based on his family history with both parents having died of an unknown condition at an early age, the absence of drug abuse, blood transfusion, or sexual intercourse and an advanced stage of the HIV infection, the most likely mode of transmission is MTCT. MTCT can occur before, during, and after (via breastfeeding) delivery [3]. The higher the maternal viral load, the higher the risk of MTCT of HIV. Intrauterine transmission is assumed to be the rarest way of vertical transmission, rating about 5–10 % when the mother is not on ART [3]. The most frequent transmission is during labour, accounting for 50–80 % in the absence of ART [4]. Postnatal transmission is estimated to be around 16 % [3]. ART during pregnancy is the best way to prevent MTCT of HIV and has importantly reduced the incidence of perinatal HIV transmission [3]. In our case, the patient's mother may not have had access to voluntary testing and ART.

Paediatric HIV infection has been shown to exert variable courses. Clinical manifestations of paediatric AIDS are different from those of adults. However, two courses have been predominantly described among children vertically infected with HIV. An early onset characterises the first type and, thus, a rapid progression of HIV infection, leading to severe clinical stages within the first year of life. The children usually suffer from *Pneumocystis jiroveci* pneumonia (PJP), encephalopathy and wasting syndromes [4]. The second type, somewhat late onset, is much slower in disease progression and is characterised by a clinical deterioration in children between five and six years old. The children may suffer from recurrent bacterial infections, generalised lymphadenopathy, hepatomegaly, lymphocytic interstitial pneumonitis, and parotitis [3], [4]. In our case, the patient had recurrent episodes of pneumonia and diarrhoea, resulting in several hospitalisations. Despite the well-documented medical history, none of the several health facilities the patient visited repeatedly over the past years offered voluntary testing for HIV. Instead, the patient was treated several times for common diseases frequently encountered in rural Gabon including malaria [5], [6].

The region and its surroundings are highly endemic for malaria, which is the most likely differential diagnosis for acute febrile episodes. The patient's symptoms had been mistaken for malaria and pneumonia for several years, with repeated treatment with several anti-infectives, including antibiotics, antimalarials and anti-helminthics, until finally establishing the correct diagnosis of AIDS with HIV-associated nephropathy. This case report reflects that HIV-positive patients risk acquiring frequent opportunistic infections and febrile illnesses due to secondary immunodeficiency. These infections and febrile illnesses in HIV-positive patients make it difficult for healthcare workers to distinguish them from diseases caused by *Plasmodium falciparum* [7] in a highly endemic region [8]. HIV-positive patients may be at greater risk of being misdiagnosed as having malaria [7]. Berg et al. have conducted a cross-sectional study where data were registered for all adults admitted to a medical ward in the Central Hospital of Maputo, Mozambique, from October to December 2006. They reported that a positive HIV status was highly associated with receiving an incorrect diagnosis for malaria. They raised the concern that antimalarials may be overused in this patient population and leave HIV patients with opportunistic infections undiagnosed and untreated [7] – a finding supported by our case report.

In our case, the patient was treated for severe malaria in the referral hospital’s paediatric ward, even though there was no evidence for *Plasmodium* spp. infection neither by RDT nor by microscopy. In line with this finding, a review reported that nearly two-thirds of a cohort of Kenyans newly infected with HIV and presenting with fever in a health facility were mistakenly treated for malaria, and only a minority was tested for HIV [9]. The repeated misdiagnosis and misuse of anti-infectives in our patients highlight several missed opportunities to avoid the rapid progression of AIDS due to the absence of ART. In addition, an important limitation for adequate patient management in a resource-limited setting, such as described in this case report, is the limited access to standard testing modalities for opportunistic infections associated with HIV infection. We were thus unable to rule out PJP nor *Cryptosporidium*-associated diarrhoea.

## Conclusions

HIV infection occurs in children and young adolescents and should be considered an important differential diagnosis of repeated febrile episodes in hyperendemic malaria regions like Gabon. Early diagnosis of HIV, particularly in children and adolescents, will help patients living with HIV by facilitating early access to ART. This case report conveys multiple learning points, among which the following are the most prominent: [1] access to HIV screening in pregnant women is an important public health intervention to avoid MTCT, and [2] HIV infection also needs to be considered in the differential diagnosis in paediatric patients foremost when presenting with a history of prolonged illness.

## Abbreviation

AIDS, Acquired Immunodeficiency Syndrome; ANC, Antenatal Care; ART, antiretroviral treatment; CDC, Centre for Disease Control; CERMEL, Centre de Recherches Médicales de Lambaréné; HBV, Hepatitis B virus; HCV, Hepatitis C virus; HIV, Human Immunodeficiency Virus; ISSA, Institut de Santé de Sindara; MTCT, Mother-to-Child Transmission; RDT, Rapid Diagnostic Test; SARS-CoV-2, Severe acute respiratory syndrome coronavirus type 2; PCR, Polymerase Chain reaction; UNAIDS, United Nations Programme on HIV/AIDS.

## CRediT authorship contribution statement

**Michael Ramharter:** Writing – review & editing, Validation, Supervision, Resources, Funding acquisition, Conceptualization. **Ayodele Alabi:** Writing – review & editing, Methodology, Investigation, Formal analysis, Conceptualization. **Saskia Dede Davi:** Writing – original draft, Project administration, Methodology, Investigation, Funding acquisition, Formal analysis, Data curation, Conceptualization. **Teite Rebecca Hildebrandt:** Investigation. **Lillian Rene Endamne:** Project administration, Investigation. **Dearie Glory Okwu:** Investigation. **Anita Lumeka:** Investigation. **Ghyslain Mombo-Ngoma:** Supervision, Resources. **Rella Zoleko-Manego:** Supervision, Resources. **Selidji Todagbe Agnandji:** Supervision, Resources.

## Ethical approval

Ethical approval for this study was given by the Institutional Ethical Committee of Centre de Recherches Médicales de Lambaréné (CEI – 002/2021).

## Consent for publication

The legal representatives of the patient provided written informed consent and the patient provided his assent to report all medically relevant data.

## Role of funding source

This research received no specific grant from funding agencies in the public, commercial, or not-for-profit sectors.

## Conflict of interest

None.

## Data Availability

The datasets generated during and analysed for this case not publicly available due to the confidentiality for the patient.=?
